# Neurodevelopmental and Behavioral Phenotypes in 14q11.2 Microduplication Syndrome: A Case Report and Literature Review

**DOI:** 10.7759/cureus.90735

**Published:** 2025-08-22

**Authors:** Joshua M Williams, Simon L Esbit, Mai-Lan Ho, Richard Sidlow

**Affiliations:** 1 Medical School for International Health, Ben Gurion University of the Negev, Be'er Sheva, ISR; 2 Radiology, University of Missouri Health Care, Columbia, USA; 3 Medical Genetics and Metabolism, Valley Children's Hospital, Madera, USA

**Keywords:** 14q11.2 microduplication syndrome, aggressive behavior, attention deficit hyperactivity disorder (adhd), autism spectrum disorder (asd), chd8, supt16h

## Abstract

An ultrarare neurodevelopmental disorder, 14q11.2 microduplication syndrome involves the *SUPT16H* and *CHD8* genes. We describe a 12-year-old male patient with a de novo 743 kb interstitial duplication detected using chromosomal microarray. He presented with a complex neurodevelopmental disorder incorporating developmental delay, intellectual disability, autism spectrum disorder, epilepsy, attention-deficit/hyperactivity disorder, obesity, aggressive behaviors, dysmorphic features, and cerebral palsy. This report adds to the limited literature on this ultrarare condition and underscores the importance of comprehensive genetic evaluation in patients with complex neurodevelopmental presentations, even in the presence of known perinatal complications.

## Introduction

An ultrarare neurodevelopmental disorder is caused by 14q11.2 microduplication, with an estimated prevalence of less than one per million people [1]. Patients with 14q11.2 microduplication and 14q11.2 microdeletion syndromes have shown a range of neurodevelopmental issues and dysmorphic features: developmental delay, intellectual disability with speech impairment, autism spectrum disorder (ASD), attention-deficit/hyperactivity disorder (ADHD), and epilepsy [2,3]. Often, there are also dysmorphic features, like hypo- and hypertelorism, dysplastic ears, short palpebral fissures, microcephaly or macrocephaly, behavioral abnormalities, stereotyped hand movements, ataxia, hypotonia, and cleft palate [3].

It has been seen that 14q11.2 microduplication and microdeletion syndromes can involve the *SUPT16H* and *CHD8* genes [2,3]. *SUPT16H* codes for a chromatin remodeling protein that interacts with histones H2A/H2B to affect nucleosome disassembly and transcription elongation [4]. *CHD8* codes for a member of the chromodomain helicase DNA binding protein family containing an SNF2-like domain and two chromatin organization-modified domains; it is also a binding partner of *CHD7*, the gene underlying CHARGE (Coloboma, Heart defects, Atresia of the choanae, Retardation of growth and development, Genital and/or urinary abnormalities, and Ear abnormalities and/or deafness) Syndrome [5,6]. Pathogenic variants in the *CHD8* gene alone have been found in patients with ASD, and pathogenic variants in *SUPT16H* alone cause a variable-expressed neurodevelopmental disorder [7].

There appears to be a phenotypic overlap between 14q11.2 microduplication syndrome, which involves the duplication of both *CHD8* and *SUPT16H*, and Prader-Willi syndrome. The latter is the result of a deletion in a region of the paternal chromosome 15 or maternal uniparental disomy or methylation differences in the same chromosome 15 region [8]. Here, we describe a case of 14q11.2 microduplication syndrome observed in a youngmale patient.

## Case presentation

A 12-year-old male patient presented to a multidisciplinary clinic initially with a chief complaint of ASD/ADHD with worsening behavioral problems, including biting, pinching, pulling wads of his own hair out, and generally more aggressive behaviors. He was born at 37.5 weeks of gestation via emergency C-section due to decreased heart rate to a 20-year-old mother and a 24-year-old father. The pregnancy was complicated by gestational diabetes mellitus, managed with insulin. The unrelated parents experienced one prior miscarriage. There was no known family history of intellectual, genetic, or developmental disorders. Birth complications included 15 minutes of resuscitation and a 49-day stay in the neonatal intensive care unit, with intubation for the first three days. He was diagnosed with hypoxic-ischemic encephalopathy. At birth, he weighed 2.61kg (5^th^ percentile) and measured 48.62cm in length (25^th^ percentile). 

**Figure 1:**
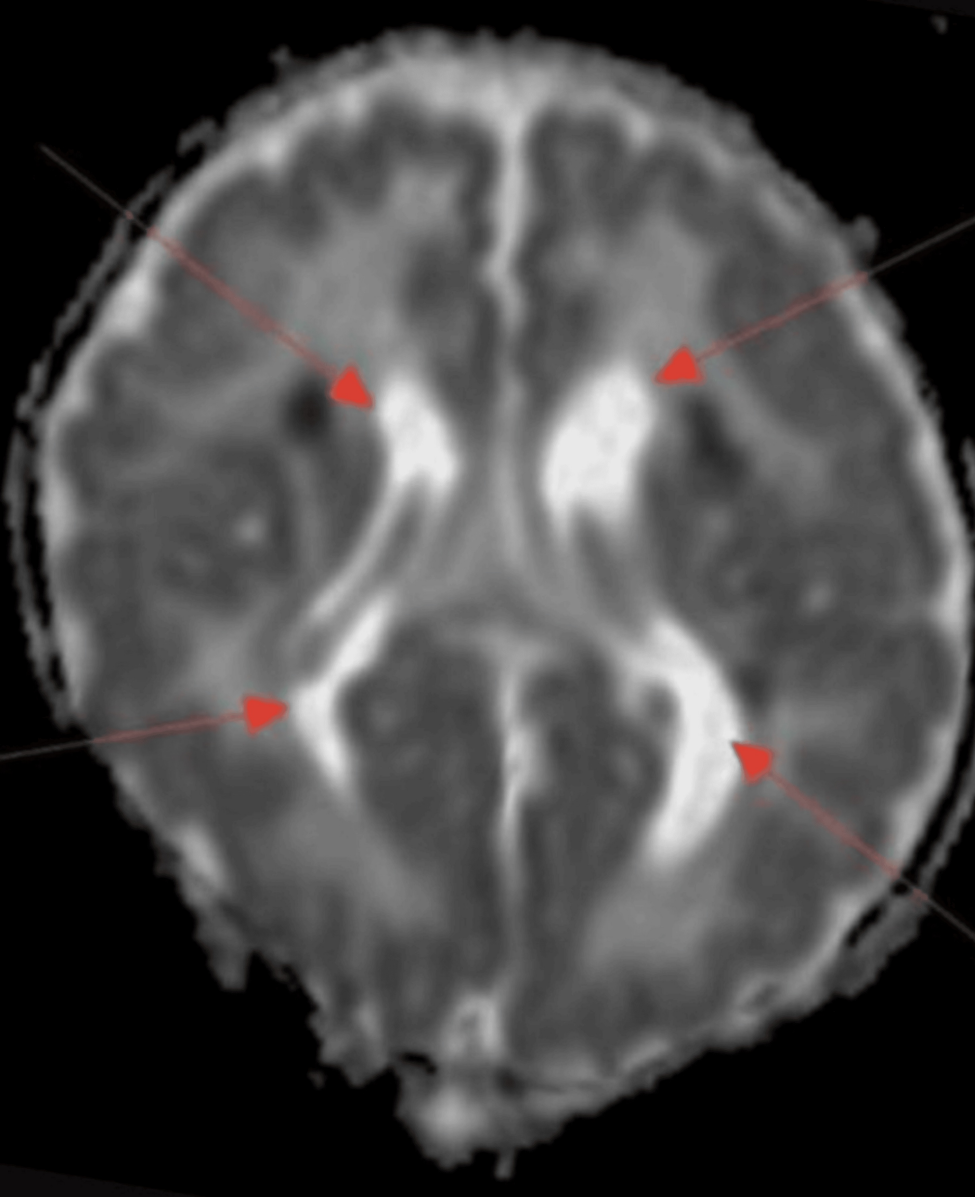
Brain MRI (axial view) showing excessive T2 hyperintense white matter (arrows) and volume loss in a periventricular distribution

**Figure 2:**
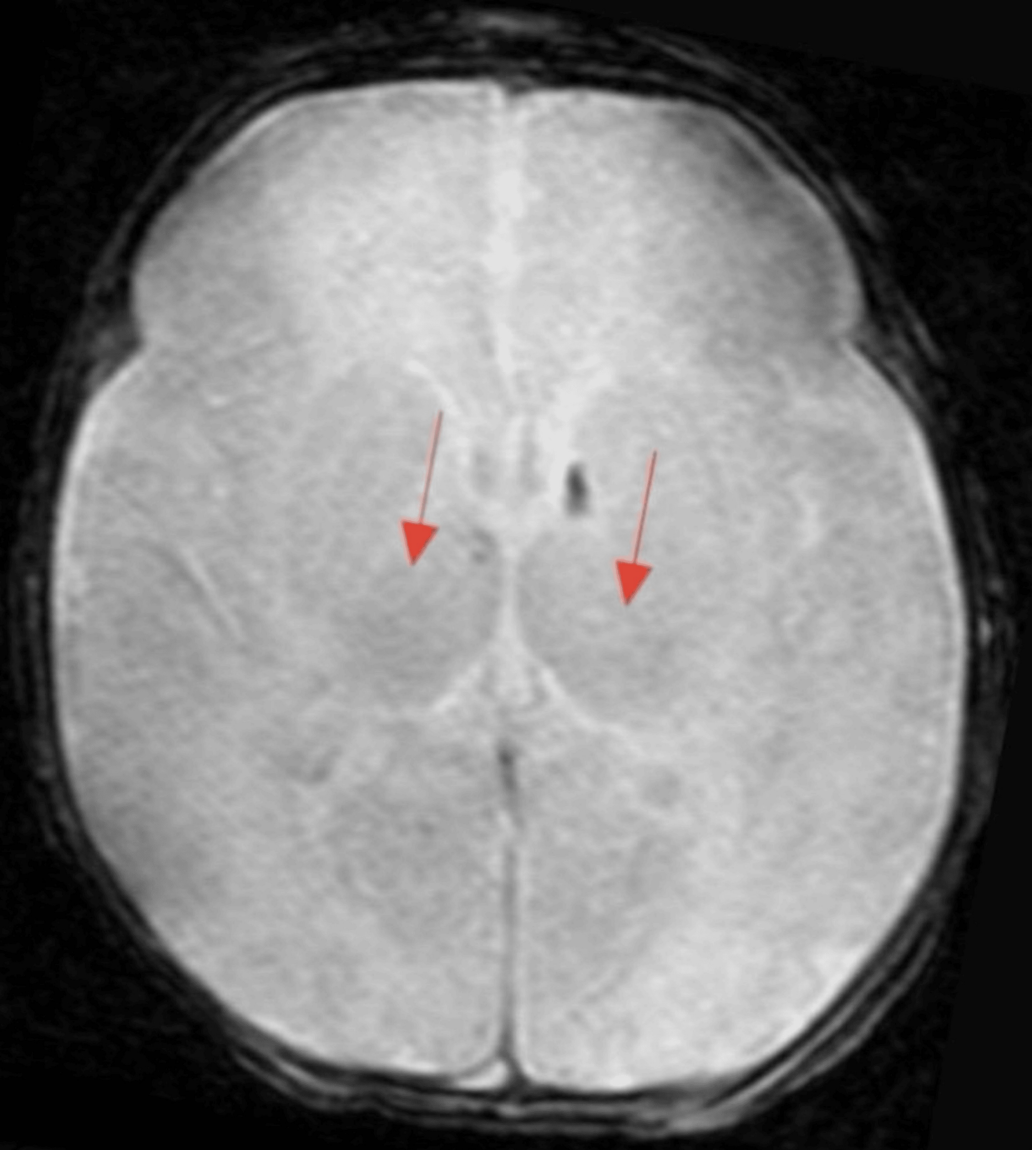
Brain MRI (axial view) showing T1 intraventricular hemorrhage (arrows)

At age two, a brain MRI showed periventricular leukomalacia with patchy T2 and fluid-attenuated inversion recovery (FLAIR) abnormalities, mild noncommunicating hydrocephalus likely due to prior intraventricular hemorrhage (IVH) (Figures 1, 2). Diagnosed with cerebral palsy (Gross Motor Function Classification System Level 2) at an age of 17 months, he displayed global mobility limitations, in addition to primary enuresis and dysphagia, the latter complicated by recurrent aspiration pneumonia, necessitating gastrostomy tube placement. 

Upon physical examination, he exhibited global hypotonia, limited ambulation, and poor postural stability characterized by a flexed-forward, mildly crouched posture. His gait was notable for an externally rotated left foot with an outward progression angle and feet in calcaneovalgus positioning. He used a wheelchair, propelling it with his feet or hands. The only dysmorphisms noted were downslanting corners of the mouth and a micropenis. 

The patient also had a history of epilepsy characterized by both focal and generalized seizures, managed with divalproex sodium, oxcarbazepine, clobazam, and brivaracetam. He was four days old when the first seizures were reported. The latest seizure-like episodes were reported at the age of 10 years. An EEG revealed abnormal intermittent interictal spikes and spike-and-wave discharges in both hemispheres (more prominent on the left than the right), with increasing frequency during drowsiness and sleep. No clinical seizures were recorded during the EEG. 

Additionally, he was diagnosed with Level 3 ASD, accompanied by restrictive and repetitive behaviors. Additional behavioral diagnoses included moderate combined-type ADHD and disruptive mood dysregulation disorder. He had an unspecified intellectual disability. At the age of four months, the Alberta Infant Motor Scale was performed, and he scored a 2 (range: 0 to 58), which is extremely low. Non-verbal, he used an augmentative and alternative communication device and attended a 5th-grade special education class with an individualized education plan. He had been in special education since starting his schooling. He could open his communication application and knew approximately seven signs. He had received occupational, physical, and speech therapy since the age of three years old and continued to do so.

The patient exhibited daily aggressive episodes (e.g., biting, pinching, destroying property, leaving bruises, and hair pulling), often directed at his mother, with increased severity in the morning, and with no apparent trigger. These episodes were accompanied by attention-seeking and repetitive behaviors, such as excessive skin picking and one occasion of ripping out his gastrostomy tube. His skin-picking behavior was managed with fluvoxamine, and lithium was used to stabilize his mood. The family employed a weighted blanket, helmet, and a designated quiet room to help him self-soothe. 

He had a history of prolonged QT syndrome, likely related to lithium, requiring periodic monitoring. Previous treatments with risperidone, chlorpromazine, aripiprazole, and divalproex sodium were discontinued due to side effects, including gynecomastia, weight gain, and concerns about prolonged QTc interval. Additional medications that were discontinued include dexmethylphenidate, clonazepam, lorazepam, and lurasidone. Dosages and schedules were unavailable for these past medications.

His current psychiatric medications included lithium (450 mg twice daily) for mood, methylphenidate (20 mg every morning, 20 mg at midday, and 15 mg at 4 pm daily) for ADHD, chlorpromazine (25 mg thrice daily) for mood and (25 mg *pro re nata* (as needed)) for aggression, fluvoxamine (100 mg twice daily for obsessive-compulsive disorder (OCD)), and clonidine (0.05 mg at 4 pm daily, 0.2 mg every night at bedtime) for ADHD and sleep. Additionally, he was on non-psychotropic medications, including brivaracetam 10mg/1mL suspension (7.5 mL twice daily) (2.48mg/kg/day), clobazam 2.5mg/1mL suspension (2mL twice daily) (0.16mg/kg/day), and oxcarbazepine 300mg/5mL suspension (10 mL twice daily).

**Figure 3:**
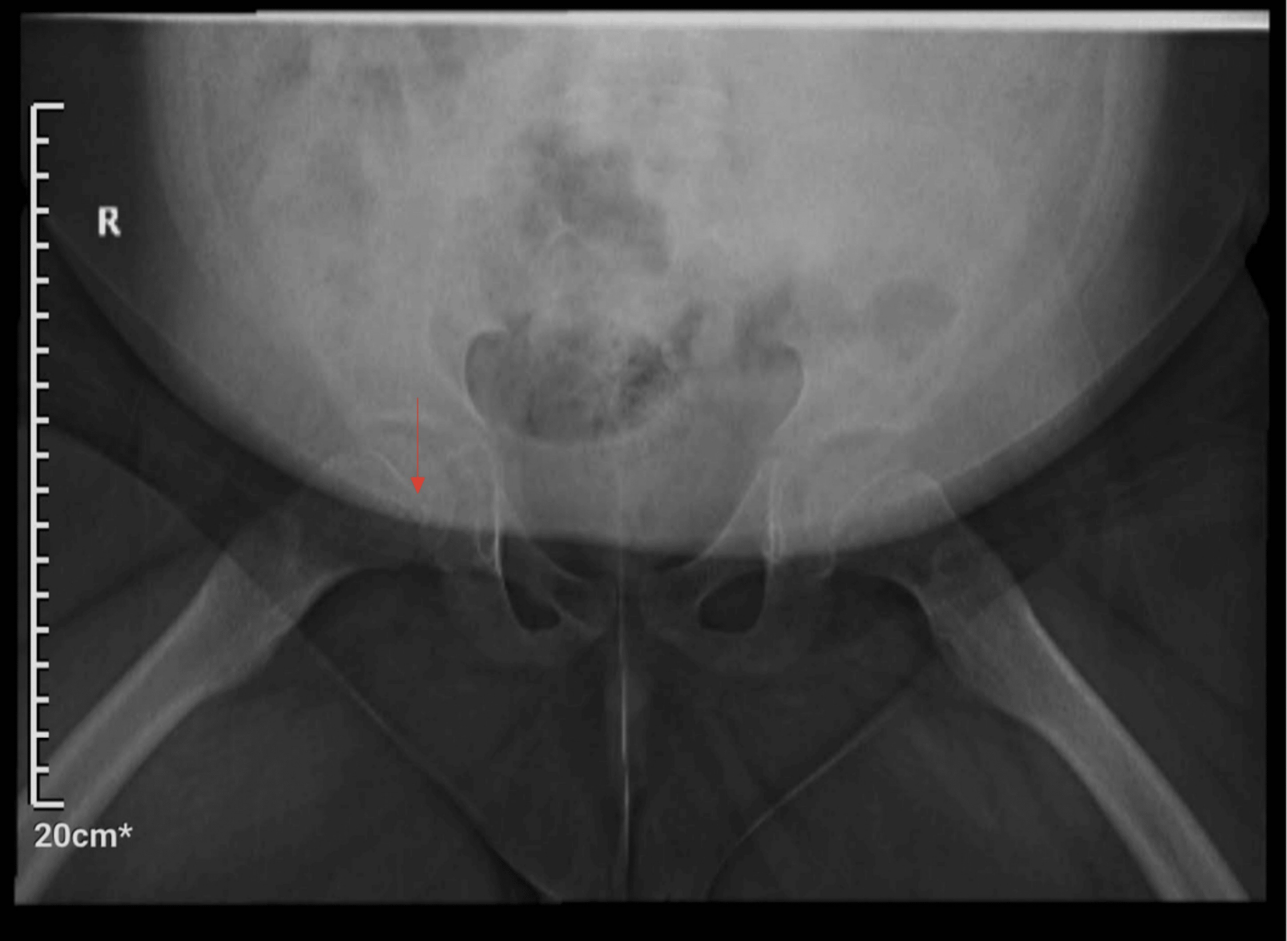
Mild asymmetric flattening of the right femoral epiphysis (arrow)

Growth tracking revealed a trajectory below, but parallel to the third percentile from ages 2 to 10, with a further decline thereafter. His bone age was 9-10 years (±10.5 months standard deviation), delayed relative to his chronological age of 11 years and 11 months. X-ray of the pelvis due to lower limb bone pain showed mild asymmetric flattening of the right femoral epiphysis, with no evidence of sclerosis or fragmentation (Figure 3). Early avascular necrosis could not be ruled out. At 12 years of age, his height was 128.3 cm (1^st^ percentile), his weight was 62.2 kg (95^th^ percentile), and his BMI was 37.79 kg/m² (99^th ^percentile). His obesity, partly attributed to chronic corticosteroid use for severe asthma, was complicated by obstructive sleep apnea with hypoxia, managed with bilevel positive airway pressure therapy. The bilevel positive airway pressure management recommendations were set at age 12, at a pressure of 12.5 cmH2O with a small AirFit N20 Nasal interface mask (ResMed Inc., San Diego, California, United States) and a heated humidifier. He also exhibited intermittent elevations in blood pressure (>130/80 mmHg), likely influenced by obesity, obstructive sleep apnea, and polypharmacy, though sustained hypertension has not been diagnosed. Echocardiography showed no left ventricular hypertrophy. Additionally, Vitamin D deficiency and elevated parathyroid hormone levels were suggestive of pseudohypoparathyroidism, managed with vitamin D. 

Surgical history included a dental procedure at age three, ankle surgery at age seven, surgical management of right hip septic arthritis and acetabular osteomyelitis at age eight, and tonsillectomy with adenoidectomy at age 10.

**Figure 4:**
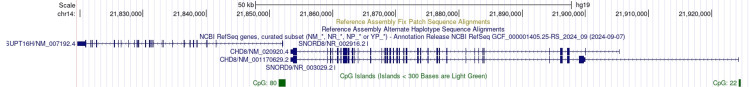
GRCh37/hg19 chr14:21,819,600-21,926,000 UCSC Genome Browser shows the presence of CpG islands neighboring the *CHD8* and *SUPT16H* genes, supporting non-allelic homologous recombination as the most likely mechanism underlying 14q11.2 microduplication syndrome [9]. CpG islands have an influence on how DNA is packaged into chromatin. CpG islands' unmethylated state promotes a more unwound and accessible chromatin structure, making it easier for transcription machinery to access the DNA [9]. UCSC: University of California, Santa Cruz

At 10 years of age, a chromosomal microarray was performed (Invitae/Labcorp Holdings Inc., Burlington, North Carolina, United States), revealing an 884.04 kb interstitial duplication at 14q11.2 [21288767_22132807]x3 [GRCh37] classified as a variant of uncertain significance (VUS). Other panel-based testing revealed carrier status for partial biotinidase deficiency (BTD c.1330G>C [p.Asp444His]) and Niemann-Pick type C (NPC1 c.3011C>T [p.Ser1004Leu]). Two VUSs in the *MTTP* gene were identified, but genotype/phenotype correlation was lacking, and phasing of these variants was not pursued. Clinical trio exome sequencing (GeneDx, Stamford, Connecticut, United States) performed at 12 years of age confirmed a heterozygous de novo pathogenic duplication of at least 743 kb at 14q11.2 [21359845_22102999]x3 [GRCh37], involving 21 genes, including *SUPT16H* and *CHD8*, which are associated with clinical disorders (Figure 4). Additionally, methylation-specific multiplex ligation-dependent probe amplification ruled out Angelman and Prader-Willi Syndromes. 

## Discussion

Twenty-five additional patients reported in the medical literature and in DECIPHER (DatabasE of Chromosomal Imbalance and Phenotype in Humans using Ensembl Resources) had similar duplications, with clinical data available for comparison alongside our patient (Table 1) [2,3,10-12]. Of the patients in Table 1, 73% (19/26), including our patient, were of the male sex. Like our patient, nine other patients had microduplications from de novo mutations, two cases were inherited maternally, and three cases were inherited paternally. Our patient, like all known patients with 14q11.2 microduplications, exhibited developmental and speech delays, and, similar to 80% of patients, had varying degrees of intellectual disability. Dysmorphic features seen in 78% of patients included brachycephaly, narrow forehead, strabismus, ptosis, short palpebral fissures, and synophrys [2,3]. Our patient demonstrated down-slanting corners of the mouth and a micropenis. A smaller proportion experienced impulsivity/ADHD (40%), aggressive behavior (53%), obesity/hyperphagia (47%), ASD (36%), and epilepsy (27%), all of which were present in our patient.

**Table 1:** 14q11.2 microduplication syndrome cases Phenotypes associated with 14q11.2 microduplication syndrome (containing CHD8 and SUPT16H). NS: not specified; NA: not applicable; CNV: copy number variation; HC: head circumference; DD: developmental delay; ID: intellectual disability; DF: dysmorphic features; + present; - absent

Study	Patient number	CNV inheritance	Sex	Age (years)	HC (at birth)	DD	Speech delay	ID	Aggressive behavior	Impulsivity/ ADHD	Autism	Obesity/ hyperphagia	Epilepsy	DF	Other findings
Current Study	NA	de novo	Male	12	NS	+	+	+ (NS)	+	+	+	+	+	+	micropenis, obstructive sleep apnea, abnormal gait, and cerebral palsy
Smol et al. [2]	Patient 1	paternal	Female	6	5^th^%ile	+	+	+ (mild)	+	+	+	–	–	–	NS
Smol et al. [2]	Patient 2	NS	Male	4	10^th^%ile	+	+	–	+	+	–	–	–	–	NS
D’Angelo et al. [10]	NA	de novo	Male	4	< 3^rd^%ile	+	+	+ (severe)	–	–	–	+	–	+	hypogonadism
Vuillaume et al. [11]	NA	maternal	Male	5	NS	+	+	NS	–	–	–	+	–	NS	NS
Smyk et al. [3]	NA	de novo	Male	8	50^th^%ile	+	+	+ (NS)	–	+	–	–	–	+	NS
Firth et al. (DECIPHER) [12]	289709	de novo	Male	22	NS	+	+	+ (NS)	–	–	–	+	–	NS	hypogonadism
Firth et al. (DECIPHER) [12]	277175	NS	Female	9	5^th^%ile.	+	+	+ (severe)	+	–	–	–	+	NS	NS
Firth et al. (DECIPHER) [12]	279247	de novo	Female	4	< 3^rd^%ile	+	+	+ (moderate)	–	–	–	–	–	+	NS
Firth et al. (DECIPHER) [12]	289620	NS	Male	53	< 3^rd^%ile	+	+	+ (moderate)	+	–	–	–	+	NS	NS
Firth et al. (DECIPHER) [12]	287656	NS	Male	11	NS	NS	NS	–	+	+	+	–	–	NS	NS
Firth et al. (DECIPHER) [12]	321788	paternal	Male	5	NS	+	+	–	–	–	+	+	–	+	NS
Firth et al. (DECIPHER) [12]	284790	de novo	Female	4	NS	NS	NS	NS	–	–	+	–	–	NS	NS
Firth et al. (DECIPHER) [12]	258583	de novo	Male	4	< 3^rd^%ile	+	+	+ (NS)	+	–	–	+	–	+	strabismus, cryptorchidism, micropenis, and hypotonia
Firth et al. (DECIPHER) [12]	303989	NS	Male	4	NS	NS	NS	NS	NS	NS	NS	NS	NS	NS	NS
Firth et al. (DECIPHER) [12]	317316	de novo	Female	2	NS	NS	NS	NS	NS	NS	NS	NS	NS	NS	NS
Firth et al. (DECIPHER) [12]	337546	NS	Male	4	NS	NS	NS	+ (NS)	NS	NS	NS	NS	NS	NS	NS
Firth et al. (DECIPHER) [12]	340128	de novo	Female	3	NS	NS	NS	NS	NS	NS	NS	NS	NS	NS	NS
Firth et al. (DECIPHER) [12]	385923	NS	Male	NS	NS	NS	NS	+ (mild)	NS	NS	NS	+	NS	+	anxiety, astigmatism, keratoconus, scoliosis, pes planus, talipes equinovarus, and poor coordination
Firth et al. (DECIPHER) [12]	390428	NS	Male	2	NS	+	NS	NS	+	NS	NS	NS	NS	NS	sleep abnormality and atypical behavior (unspecified)
Firth et al. (DECIPHER) [12]	429153	paternal	Male	16	NS	NS	NS	+ (mild)	NS	+	NS	NS	NS	NS	specific learning disability (unspecified)
Firth et al. (DECIPHER) [12]	476723	NS	Male	NS	NS	NS	NS	NS	NS	NS	NS	NS	NS	NS	NS
Firth et al. (DECIPHER) [12]	481505	NS	Female	8	NS	NS	NS	NS	NS	NS	NS	NS	NS	NS	NS
Firth et al. (DECIPHER) [12]	501059	NS	Male	NS	NS	+	NS	NS	NS	NS	NS	NS	+	NS	psychosis
Firth et al. (DECIPHER) [12]	510044	maternal	Male	NS	NS	NS	+	NS	NS	NS	NS	NS	NS	NS	atypical behavior (unspecified)
Firth et al. (DECIPHER) [12]	510371	NS	Male	5	NS	NS	NS	NS	NS	NS	NS	NS	NS	NS	NS
Total n = 26		NA	(19/26) 73% Male	NA	NA	14/14 (100%)	13/13 (100%)	12/15 (80%)	8/15 (53%)	6/15 (40%)	5/14 (36%)	7/15 (47%)	4/15 (27%)	7/9 (78%)	NA

In comparison to other duplications and deletions in the same region, the minimal duplicated/deleted region is the 35 kb locus containing the *CHD8* and *SUPT16H* genes, and this is consistent with the fact that variants within and deletion of this chromosomal interval also cause neurodevelopmental deficits such as developmental delay, intellectual disability with speech impairment, ASD, ADHD, and epilepsy [2,3]. Common dysmorphic features included hypertelorism, down-slanting palpebral fissures, a broad nose, an elongated philtrum, a pronounced Cupid’s bow of the upper lip, a full lower lip, and auricular anomalies. Additionally, macrocephaly was reported in 75% of cases involving microdeletions and truncating pathogenic variants [2,3]. *CHD8 *gene mutations in other disorders, such as *CHD8*-related neurodevelopmental disorder with overgrowth, have been implicated in ASD, intellectual disability, developmental delay, neuropsychiatric issues, neurologic problems, sleep disturbance, and gastrointestinal issues [13]. It can present with physical features such as macrocephaly, usually in infancy, and tall stature, usually during puberty [13]. Similarly, de novo variants in *SUPT16H *have been shown to play a role in a variety of symptoms in patients, such as intellectual disability, autistic features, precocious puberty, sleeping difficulties, and seizures [14]. There were also dysmorphic features displayed, like a tall forehead, down-slanting palpebral fissures, ear anomalies, and a broad nasal bridge [14].

As shown in Figure 4, the UCSC Genome Browser illustrates CpG islands neighboring both *SUPT16H* and *CHD8* [9]. Notably, this region is included in all cases of 14q11.2 microduplication syndrome documented in Table 1, supporting non-allelic homologous recombination as the most likely mechanism underlying this phenomenon. ClinGen (The Clinical Genome Resource) argues against triplosensitivity in 14q11.2 microduplication syndrome [15]. However, the cases enumerated in Table 1 of the study suggest that duplications involving the *CHD8* and *SUPT16H* genes argue otherwise.

While there are clinical similarities between 14q11.2 microduplication syndrome and Prader-Willi syndrome, sharing features such as hyperphagia/obesity, hypogonadism, developmental delay, and aggressive behavior, this diagnostic possibility was ruled out in our patient via genetic testing [8]. Many of the neurodevelopmental aspects of our case (e.g., aggressive behavior, complex motor phenotype without spasticity, speech difficulties) cannot necessarily be ruled out as sequelae of the hypoxic-ischemic encephalopathy experienced perinatally [16]. Concomitantly, it cannot be ruled out that our patient’s perinatal course was not a part of the 14q11.2 duplication syndrome itself. Our patient's presentation may represent a case of dual pathology, in which both the perinatal complications (hypoxic-ischemic encephalopathy (HIE) and cerebral palsy) and the 14q11.2 microduplication syndrome contribute to the patient’s overall clinical picture. This case highlights the need for thorough genetic assessment in children with neurodevelopmental delays and atypical phenotypes, even when perinatal complications are present, to ensure accurate diagnosis, counseling, and management.

Currently, there is no known cure for 14q11.2 microduplication syndrome, and treatment focuses on symptom management, such as speech therapy, occupational therapy, behavioral therapy, anti-epileptic pharmacological therapy, etc [3]. As a future direction, establishing a dedicated foundation or support network for 14q11.2 microduplication syndrome could benefit affected individuals and their families by fostering connection, providing shared resources, and supporting advocacy efforts for research, clinical guidance, and public awareness [1,3].

## Conclusions

This case report highlights the complex presentation of a patient with 14q11.2 microduplication syndrome involving the *SUPT16H* and *CHD8 *genes. While many clinical features, such as developmental delay, intellectual disability, ADHD, ASD, obesity, and dysmorphic facial features, are consistent with previously reported cases of 14q11.2 microduplication syndrome, this patient’s history of HIE and cerebral palsy may contribute to their motor deficits. This case underscores the importance of comprehensive genetic evaluation in children with neurodevelopmental delays and atypical phenotypes, even in the presence of known perinatal complications, to ensure accurate diagnosis, counseling, and management.
